# Effects of Danshen Ethanol Extract on the Pharmacokinetics of Fexofenadine in Healthy Volunteers

**DOI:** 10.1155/2014/473213

**Published:** 2014-11-03

**Authors:** Furong Qiu, Jin Zeng, Songcan Liu, Min He, Leilei Zhu, Yujie Ye, Ping Miao, Shujiao Shen, Jian Jiang

**Affiliations:** Laboratory of Clinical Pharmacokinetics, Shuguang Hospital, Shanghai University of Traditional Chinese Medicine, Shanghai 201203, China

## Abstract

This study investigated the effect of multidose administration of danshen ethanol extract on fexofenadine pharmacokinetics in healthy volunteers. A sequential, open-label, two-period pharmacokinetic interaction design was used. 12 healthy male volunteers received a single oral dose of fexofenadine (60 mg) followed by danshen ethanol extract (1 g orally, three times a day) for 10 days, after which they received 1 g of the danshen extract with fexofenadine (60 mg) on the last day. The plasma concentrations of fexofenadine was measured by LC-MS/MS. After 10 days of the danshen extract administration, the mean AUC and *C*_max⁡_ of the fexofenadine was decreased by 37.2% and 27.4% compared with the control, respectively. The mean clearance of fexofenadine was increased by 104.9%. The *in vitro* study showed that tanshinone IIA and cryptotanshinone could induce MDR1 mRNA. This study showed that multidose administration of danshen ethanol extract could increase oral clearance of fexofenadine. The increased oral clearance of fexofenadine is attributable to induction of intestinal P-glycoprotein.

## 1. Introduction

Herb used as complementary and alternative medicine (CAM) has dramatically increased over the last 20 years. Due to the widespread use of CAM in combination with proprietary medications, there is a strong possibility of herb-drug interactions (HDIs) involving absorption and/or metabolism and/or excretion processes. Recent progress in the study of membrane transport has expanded our understanding of the mechanisms underlying pharmacokinetic HDIs involving transporters [1].

The extract from the roots of* Salvia miltiorrhiza* (danshen) is widely and traditionally used in the treatment of angina pectoris, myocardial infarction, stroke, and cancer in China and other countries [2–5]. The commercially available preparations from danshen extract are primarily formulated with the ethanol extract, in which the diterpenoid tanshinones accounted for approximately 95% of the total amount with cryptotanshinone, tanshinone IIA, and tanshinone I as the major components [6]. We found that danshen ethanol extract could induce CYP3A4* in vivo* [6], and the two major components, cryptotanshinone and tanshinone IIA, present in the extract are responsible for CYP3A4 induction via the activation of PXR [7]. Because CYP3A4 and MDR1 genes have PXR transcriptional binding sites and common molecular mechanism responsible for induction of CYP3A4 and MDR1 by ligand, cryptotanshinone and tanshinone IIA may be assumed to induce MDR1 (also called P-glycoprotein, P-gp) [8].

Currently there is little knowledge about whether the danshen extract has a modulatory effect on human* in vivo* P-gp. The aim of this study was to investigate multidose administration of danshen ethanol extract on* in vivo* MDR1 activity in healthy volunteers. The constituent(s) *r* induced to MDR1 was also investigated using human cryopreserved hepatocytes. It will provide valuable information for using the danshen preparation in clinical practice.

## 2. Methods

### 2.1. Study Drugs

The danshen ethanol extract in the form of capsule (250 mg/capsule, Lot 20090904) was manufactured, and the quality control was established and enforced strictly by Hebei Xinlong XiLi Pharmaceuticals Ltd. according to state drug standard (China State Food and Drug Administration, Ws3-B-3140-98-009). The contents of tanshinone IIA, cryptotanshinone, and tanshinone I were 106.2 mg/g, 88.0 mg/g, and 53.1 mg/g, respectively [6]. Fexofenadine tablets (60 mg/tablet, Lot 100827) were manufactured by Jiangsu Hengrui Pharmaceuticals Ltd.

### 2.2. Subjects and Ethical Approval

Twelve male healthy Chinese volunteers participated in this study (age range, 25–30 years; BMI range, 19–25 kg/m^2^). These volunteers were enrolled in the study after obtaining written informed consent. The clinical protocol and informed consent form were approved by the independent medical ethics committee of Shuguang Hospital affiliated with the Shanghai University of Traditional Chinese Medicine.

All subjects were nonsmokers and were judged to be healthy by a medical history, a physical examination, electrocardiogram, and laboratory tests (including complete blood count, blood biochemistry testing, and urinalysis) before entering the study. Subjects abstained from consuming herbal and citrus fruit products for 2 weeks before the study and from alcohol and medications for 2 weeks before and during the study period, and caffeine-containing foods, orange juice, grapefruit juice, and beverages were also excluded during the study period.

### 2.3. Study Design

The study design was a sequential, open-label, two-period trial conducted at the Shuguang Hospital phase I clinical trial ward [6]. On the morning of day 1 the volunteers took a single dose of 60 mg of fexofenadine. Beginning on day 2, they received the danshen extract (1 g, three times a day) for 10 days. On day 12, the volunteers received 1 g of the danshen extract together with 60 mg of fexofenadine. The volunteers fasted overnight before each dosing. The subjects were provided a light standard meal at 4 h after medication intake and at 6 p.m. on the two test days of intaking probe drugs. Blood samples (4 mL each) were drawn before and at 0.25, 0.5, 1, 1.5, 2, 3, 4, 6, 8, 10, 12, and 24 hours after fexofenadine administration and kept in heparinized Eppendorf tubes. The blood samples were centrifuged, and plasma was separated and stored at −80°C until the time of analysis.

### 2.4. Sample Analysis

Plasma samples were spiked with an internal standard (diazepam) and extracted with ethyl acetate. After evaporation of the organic solvent under nitrogen, reconstituted residues of the organic phase were analyzed using a Sciex API 4000 coupled liquid chromatography-tandem mass spectrometry (LC-MS/MS) system (Applied Biosystems/SCIEX, CA). Chromatographic separation of the compounds was accomplished using a C_18_ column (5 *μ*m, 4.6 mm × 150 mm, Agilent, America) with water phase (ammonium acetate 4 mmol/L and methanoic acid 0.08%) : methanol (10 : 90, v : v) as the mobile phase at a flow rate of 0.80 mL/min. The mass spectrometer was operated in the MRM mode under positive ionization. The ion transitions monitored were mass-to-charge ratios of 502.2–466.5 (fexofenadine) and 285.2–193.1 (internal standard). The collision energy (CE), declustering potential (DP), and collision cell exit potential (CXP) were set as follows: fexofenadine: 36.32 V, 100.32 V, and 16.09 V, respectively; diazepam: 43.00 V, 98.06 V, and 11.45 V, respectively. A set of 7 nonzero calibration standards, ranging from 1 to 500 ng/mL, and 3 quality controls at concentrations of 2, 50, and 400 ng/mL were performed during each day of analysis. The interday CVs for the low, middle, and high quality controls were 1.67%, 3.34%, and 3.10%, respectively. The intraday CVs were 3.36%, 12.84%, and 0.33%, respectively. The recovery for the low, middle, and high quality controls was 100.91%, 96.11%, and 92.11%, respectively.

### 2.5. Induction of MDR1 mRNA by Tanshinones in Human Hepatocytes

Cryopreserved human hepatocytes (Lot ONO and JYM), provided by the Research Institute for Liver Disease Co. (Shanghai, China), were thawed in plating medium and transferred to collagen I precoated 24-well plates at a density of 3.0 × 10^5^ viable cells. The cells were maintained at 37°C in a humidified incubator with 90% atmospheric air and 5% CO_2_. After the initial 24-hour conditioning culture, the medium was removed and the hepatocytes were treated with vehicle, which contained the same amount of DMSO (0.1%), tanshinones (2 *μ*M, 10 *μ*M), and rifampicin (25 *μ*M) for 72 hours. All drugs were dissolved in DMSO and then added to the culture medium (final DMSO concentration, 0.1%). Total RNA was isolated from cells using TRIzol reagent (Invitrogen) according to the manufacturer-supplied protocol. Quantitative real-time PCR was performed using gene-specific primers and the SYBR Green PCR kit (Invitrogen) in an ABI 7900 system (Applied Biosystems). The relative quantity of the target MDR1 gene compared with the endogenous control (glyceraldehyde-3-phosphate dehydrogenase) was determined by the ΔΔCT method. The following primer sets were used in this study: MDR1 (5′CGGACATCCCAGTGCTTCA-3′ and 5′-GTCGCTTTATTTCTTTGCCATC-3′).

### 2.6. Pharmacokinetic Determination

The plasma concentration-time data of analyteswere analyzed by compartment-independent approaches. The maximum plasma drug concentration (*C*_max⁡_) and time to *C*_max⁡_ (*T*_max⁡_) were directly obtained from the plasma concentration-time data. The elimination half-life (*t*_1/2_) was calculated as 0.693/Ke, where Ke, the elimination rate constant, was calculated via semilog regression on the terminal phase of the plasma concentration-time curve. The AUC from time 0 to infinity (AUC_0−*∞*_) was estimated as AUC_0−*t*_ + *C*_*t*_/Ke, where *C*_*t*_ is the plasma concentration of the last measurable sample and AUC_0−*t*_ was calculated according to the linear trapezoidal rule. Total plasma clearance (CL/F) was calculated as dose/AUC_0−*∞*_.

### 2.7. Statistical Analysis

Statistical comparisons between control and drug treatments were initially performed by two-way ANOVA for repeated measures performed with SAS for Windows software (version 9.2; SAS Institute Inc.). For the ANOVA analyses of treatment effect with *P* < 0.05,* post hoc* a priori comparisons were performed between control and the treatment with the paired *t*-tests. *T*_max⁡_ was analyzed using Wilcoxon's signed rank test. *P* < 0.05 indicated statistical significance. 90% confidence intervals (CIs) were constructed for the ratios of with- to without-danshen treatment using the log-transformed data for the geometric means of *C*_max⁡_, AUC_0–24_, AUC_0–*∞*_, *t*_1/2_, and CL/F. The resulting confidence limits were transformed by exponentiation and reported on the original measurement scale. The statistical limits were set at 0.80–1.25. The DAS statistical analysis system (version 1.0) was also used.

## 3. Results

No clinically undesirable signs or symptoms attributable to the administration of fexofenadine and the danshen extract were recognized during the study. All subjects completed the study according to the protocol.

**Figure 1:**
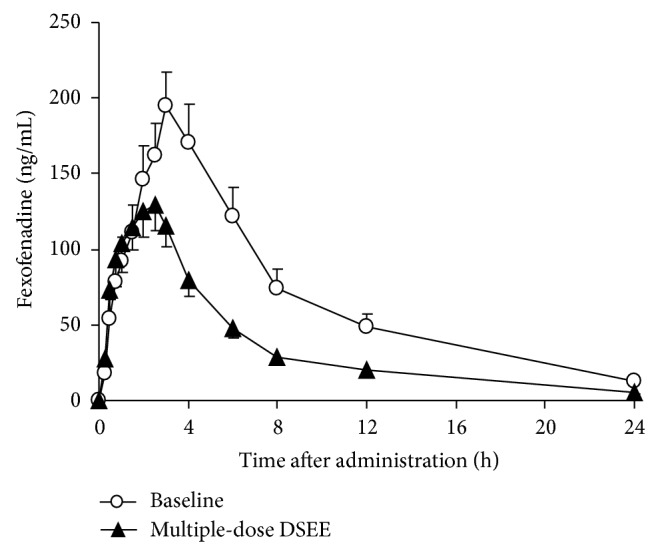
Mean (±SE, *n* = 12) plasma concentration of fexofenadine after the administration of a single dose of 60 mg of fexofenadine before and after multiple-dose coadministration of danshen ethanol extract.

The mean plasma concentration-time profiles of fexofenadine before and after multidose administration of the danshen extract are presented in Figure 1.  Table 1 summarizes the pharmacokinetic parameters of fexofenadine before and after multidose of the danshen extract treatment.

**Figure 2:**
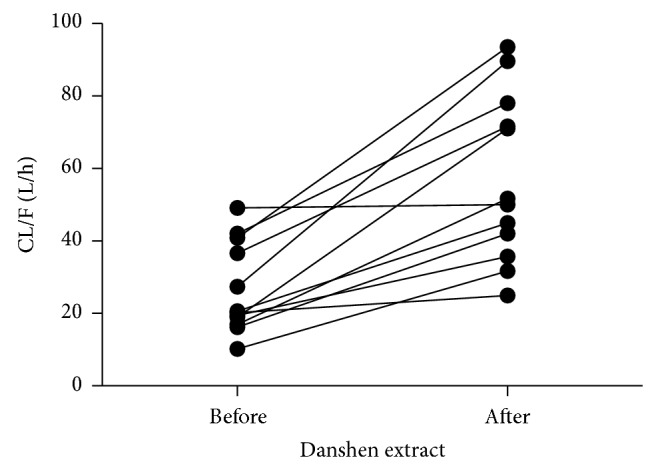
Individual value of oral clearance for fexofenadine before and after multidose of the danshen extract treatment (*n* = 12).

After 10 days of danshen ethanol extract administration, AUC of fexofenadine was decreased by 37.2% (1612.23 ± 773.58 ng·h/mL versus 844.57 ± 339.67 ng*·*h/mL) compared with the control. The clearance of fexofenadine was increased by 104.9% (44.35 ± 25.57 L/h versus 77.88 ± 31.20 L/h), and 12 volunteers' individual data of CL/F are shown in Figure 2. The *C*_max⁡_ was decreased by 27.4% (223.92 ± 74.36 ng/mL versus 148.05 ± 60.93 ng/mL) compared with the control. 90% CIs of *C*_max⁡_ and AUC_0–*∞*_ of fexofenadine were under low limit of bioequivalence (<0.80). 90% CIs of CL/F of fexofenadine were beyond upper limit of bioequivalence (>1.25). But 90% CIs of *t*_1/2_ were within range of bioequivalence (0.80–1.25). A Wilcoxon signed rank test for fexofenadine indicated that *T*_max⁡_ was nonsignificant difference (*P* > 0.05).

**Figure 3:**
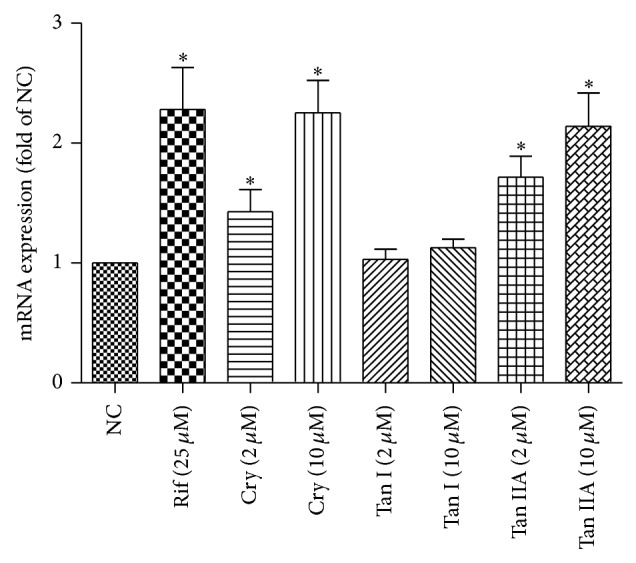
Induction of MDR1 mRNA by tanshinone I (TanI), cryptotanshinone (Cry), and tanshinone IIA (Tan IIA). Human hepatocytes were exposed to tanshinone I (2 *μ*M, 10 *μ*M), cryptotanshinone (2 *μ*M, 10 *μ*M), tanshinone IIA (2 *μ*M, 10 *μ*M), or 25 *μ*M rifampin (PC) for 3 days. MDR1 mRNA levels were measured with reverse transcription real-time PCR. These data were obtained from two independent experiments, and each experiment was performed in triplicate. Each column with bar represents the mean and S.D. The mean is expressed as fold induction over vehicle control (NC).

The effects of the tanshinones on MDR1 mRNA were shown in Figure 3. Rifampicin can upregulate MDR1 transcripts 2.28-fold at 25 *μ*M. Cryptotanshinone increased MDR1 transcripts by 1.43- and 2.25-fold at 2 *μ*M and 10 *μ*M, respectively. Tanshinone IIA increased MDR1 transcripts by 1.72- and 2.14-fold at 2 *μ*M and 10 *μ*M, respectively. Tanshinone I did not increase MDR1 transcripts (1.03- and 1.13-fold) at 2 *μ*M and 10 *μ*M.

## 4. Discussion

This study examined the effect of multiple doses of danshen ethanol extract on the pharmacokinetics of fexofenadine in healthy subjects. To our knowledge, this is the first report to evaluate the effect of danshen ethanol extract on fexofenadine in healthy volunteers.

Fexofenadine, an orally active nonsedating H_1_-receptor antagonist prescribed for oral treatment of allergic rhinitis and chronic idiopathic urticaria has been shown to be a substrate of P-glycoprotein (P-gp) [9–11]. MDR1 inhibitors (itraconazole, ritonavir, and verapamil) can increase while inducers (carbamazepine, rifampicin, and St John's wort) decrease fexofenadine AUC and *C*_max⁡_ [12–18]. Furthermore, it had good clinical safety over a wide dose range. So, fexofenadine was possibly a superior clinical drug probe to evaluate the effect of the danshen extract on MDR1 activity. Although fexofenadine is a widely used probe drug of MDR1 activity* in vivo*, which mediated the cellular efflux of fexofenadine to reduce the absorption of its substrates [9–13], this nonsedating antihistamine drug is also known to be a substrate of drug uptake transporters. Among the investigated uptake transporters, OATP1A2 was the only OATP transporter capable of fexofenadine uptake in intestine [19]. OATP1A2 colocalized with MDR1 to the brush border domain of enterocytes. Unlike P-glycoprotein, enteric OATP acts to facilitate the absorption of its substrates [19].

This study showed that multiple doses of the danshen extract significantly increased the oral clearance (CL/F) and decreased the bioavailability of fexofenadine without affecting the terminal half-life. Because of a negligible hepatic first-pass effect, the bioavailability of fexofenadine is attributable to its absorption in the intestine. Given that the danshen extract reduces the bioavailability of fexofenadine, the induction of intestinal efflux (MDR1) can be the underlying mechanism. The* in vivo* findings do not support induction of OATP1A2 because this may contribute to increasing the bioavailability of fexofenadine, not to decreasing the bioavailability. But inhibition of the danshen extract to OATP1A2 was not supported because single dose administration of the danshen extract can increase systemic drug exposure of fexofenadine (data not shown). So, the danshen ethanol extract was found to markedly reduce the systemic exposure of fexofenadine, mainly via the induction of intestinal MDR1 proteins that led to enhanced clearance.

The induction of CYP3A4 and MDR1 is thought to be mediated by the nuclear receptor PXR. PXR is predominantly expressed in the human liver and intestine [20, 21], so, the* in vitro* induction studies were conducted in cryopreserved human hepatocytes as reported previously [22] to evaluate the relevance of the induction of MDR1 by the danshen extract. In* in vitro* studies we found that tanshinone IIA and cryptotanshinone could induce MDR1 mRNA using the primary human hepatocytes. The induction of MDR1 by tanshinone IIA and cryptotanshinone at 10 *μ*M can be comparable to the effect of rifampin at 25 *μ*M.

We also found that danshen ethanol extract could induce CYP3A in healthy Chinese volunteers [6]. MDR1 and CYP3A together constitute a highly efficient barrier for many orally absorbed drugs which are substrates for both P-gp and CYP3A4 [23, 24]. This synergistic effect of danshen ethanol extract is more evident for cosubstrates for P-gp and CYP3A4.

## Figures and Tables

**Table 1:** Pharmacokinetic parameters of fexofenadine after a single dose administration of 60 mg fexofenadine in 12 healthy volunteers before and after multiple-dose coadministration of danshen ethanol extract.

PK parameter	Control	Multiple dose	Ratio to control (multiple doses)
*C*_max⁡_ (ng/mL)	223.92 ± 74.36	148.05 ± 60.93^*^	0.65 (0.51–0.83)
*T*_max⁡_ (h)	3 (1.5–6.0)	2.0 (0.5–3.0)	—
AUC_0–24_ (ng*·*h/mL)	1612.23 ± 773.58	844.57 ± 339.67^*^	0.55 (0.42–0.71)
AUC_0–∞_ (ng*·*h/mL)	1717.66 ± 815.21	889.99 ± 353.11^*^	0.54 (0.41–0.70)
*T*_1/2_ (h)	5.97 ± 0.82	5.96 ± 1.00	0.99 (0.89–1.11)
CL/F (L/h)	44.35 ± 25.57	77.88 ± 31.20^*^	1.86 (1.43–2.42)

Data are presented as mean ± SD. Data of ratio to control are shown as mean (95% confidence interval); *T*_max⁡_ data are shown as median (range).

^*^ *P* values are given for the differences with respect to control.

## References

[1] Giacomini K. M., Huang S.-M., Tweedie D. J., Benet L. Z., Brouwer K. L. R., Chu X., Dahlin A., Evers R., Fischer V., Hillgren K. M., Hoffmaster K. A., Ishikawa T., Keppler D., Kim R. B., Lee C. A., Niemi M., Polli J. W., Sugiyama Y., Swaan P. W., Ware J. A., Wright S. H., Wah Yee S., Zamek-Gliszczynski M. J., Zhang L. (2010). Membrane transporters in drug development. *Nature Reviews Drug Discovery*.

[2] Cheng T. O. (2006). Danshen: A popular chinese cardiac herbal drug. *Journal of the American College of Cardiology*.

[3] Xu Y. Y., Wan R. Z., Lin Y. P., Yang L., Chen Y., Liu C. X. (2007). Recent advance on research and application of Salvia miltiorrhiza. *Asian Journal of Drug Metabolism and Pharmacokinetics*.

[4] Ji X. Y., Tan B. K. H., Zhu Y. Z. (2000). Salvia miltiorrhiza and ischemic diseases. *Acta Pharmacologica Sinica*.

[5] Song M., Hang T.-J., Zhang Z., Chen H.-Y. (2007). Effects of the coexisting diterpenoid tanshinones on the pharmacokinetics of cryptotanshinone and tanshinone IIA in rat. *European Journal of Pharmaceutical Sciences*.

[6] Qiu F., Jiang J., Ma Y., Wang G., Gao C., Zhang X., Zhang L., Liu S., He M., Zhu L., Ye Y., Li Q., Miao P. (2013). Opposite effects of single-dose and multidose administration of the ethanol extract of danshen on CYP3A in healthy volunteers. *Evidence-Based Complementary and Alternative Medicine*.

[7] Yu C., Ye S., Sun H., Liu Y., Gao L., Shen C., Chen S., Zeng S. (2009). PXR-mediated transcriptional activation of CYP3A4 by cryptotanshinone and tanshinone IIA. *Chemico-Biological Interactions*.

[8] Geick A., Eichelbaum M., Burk O. (2001). Nuclear receptor response elements mediate induction of intestinal MDR1 by rifampin. *Journal of Biological Chemistry*.

[9] Perloff M. D., von Moltke L. L., Greenblatt D. J. (2002). Fexofenadine transport in Caco-2 cells: inhibition with verapamil and ritonavir. *Journal of Clinical Pharmacology*.

[10] Drescher S., Schaeffeler E., Hitzl M., Hofmann U., Schwab M., Brinkmann U., Eichelbaum M., Fromm M. F. (2002). *MDR1* gene polymorphisms and disposition of the P-glycoprotein substrate fexofenadine. *British Journal of Clinical Pharmacology*.

[11] Zhou Q., Ye Z., Ruan Z., Zeng S. (2013). Investigation on modulation of human *P*-gp by multiple doses of Radix Astragali extract granules using fexofenadine as a phenotyping probe. *Journal of Ethnopharmacology*.

[12] Garrett M., Smeraglia J., Lin X., Tan L. (2005). A pilot study to assess simultaneous administration of oral midazolam (MDZ) and fexofenadine (FEX) for the evaluation of cytochrome (CYP) 3A4 and P-glycoprotein (P-GP) activities. *Clinical Pharmacology & Therapeutics*.

[13] Shimizu M., Uno T., Sugawara K., Tateishi T. (2006). Effects of single and multiple doses of itraconazole on the pharmacokinetics of fexofenadine, a substrate of P-glycoprotein. *British Journal of Clinical Pharmacology*.

[14] van Heeswijk R. P. G., Bourbeau M., Campbell P., Seguin I., Chauhan B. M., Foster B. C., Cameron D. W. (2006). Time-dependent interaction between lopinavir/ritonavir and fexofenadine. *Journal of Clinical Pharmacology*.

[15] Sakugawa T., Miura M., Hokama N., Suzuki T., Tateishi T., Uno T. (2009). Enantioselective disposition of fexofenadine with the P-glycoprotein inhibitor verapamil. *British Journal of Clinical Pharmacology*.

[16] Akamine Y., Miura M., Yasui-Furukori N., Kojima M., Uno T. (2012). Carbamazepine differentially affects the pharmacokinetics of fexofenadine enantiomers. *The British Journal of Clinical Pharmacology*.

[17] Hamman M. A., Bruce M. A., Haehner-Daniels B. D., Hall S. D. (2001). The effect of rifampin administration on the disposition of fexofenadine. *Clinical Pharmacology & Therapeutics*.

[18] Wang Z., Hamman M. A., Huang S.-M., Lesko L. J., Hall S. D. (2002). Effect of St John's wort on the pharmacokenetics of fexofenadine. *Clinical Pharmacology and Therapeutics*.

[19] Shimizu M., Fuse K., Okudaira K., Nishigaki R., Maeda K., Kusuhara H., Sugiyama Y. (2005). ontribution of OATP (organic anion-transporting polypeptide) family transporters to the hepatic uptake of fexofenadine in humans. *Drug Metabolism and Disposition*.

[20] Nishimura M., Naito S., Yokoi T. (2004). Tissue-specific mRNA expression profiles of human nuclear receptor subfamilies. *Drug Metabolism and Pharmacokinetics*.

[21] Nishimura M., Naito S. (2005). Tissue-specific mRNA expression profiles of human ATP-binding cassette and solute carrier transporter superfamilies. *Drug Metabolism and Pharmacokinetics*.

[22] Williamson B., Dooley K. E., Zhang Y., Back D. J., Owen A. (2013). Induction of influx and efflux transporters and cytochrome P450 3A4 in primary human hepatocytes by rifampin, rifabutin, and rifapentine. *Antimicrobial Agents and Chemotherapy*.

[23] Pal D., Mitra A. K. (2006). MDR- and CYP3A4-mediated drug-herbal interactions. *Life Sciences*.

[24] Dresser G. K., Schwarz U. I., Wilkinson G. R., Kim R. B. (2003). Coordinate induction of both cytochrome P4503A and MDR1 by St John's wort in healthy subjects. *Clinical Pharmacology and Therapeutics*.

